# Supplementary material for: Impact of blood meals taken on ivermectin-treated livestock on survival and egg production of the malaria vector Anopheles coluzzii under laboratory conditions
Source: PLoS One. 2024 Aug 15;19(8):e0308293. doi: 10.1371/journal.pone.0308293 (PMC11326554; doi:10.1371/journal.pone.0308293)
Supplement: S1 Table — (PDF) [file pone.0308293.s001.pdf]

Supporting S1 Table: Number of exposed, blood-fed and dissected female *Anopheles* according to the hosts species, the treatment and the time elapsed since the injection (DAI)

**Hosts species**	**DAI**	**Control**					**Therapeutic dose**					**Double therapeutic dose**					**Triple therapeutic dose**				
		**T1**	**G1**	**T2**	**G2**	**Diss.**	**T1**	**G1**	**T2**	**G2**	**Diss.**	**T1**	**G1**	**T2**	**G2**	**Diss.**	**T1**	**G1**	**T2**	**G2**	**Diss.**
Sheep		Ivermectin : 0 mg/kg					Ivermectin at 0.2 mg/kg														
	Before	472	443	292	121	71	512	471	274	132	99	-	-	-	-	-	-	-	-	-	-
	2 DAI	445	209	138	101	47	555	356	56	34	34	-	-	-	-	-	-	-	-	-	-
	7 DAI	411	205	100	91	60	345	246	90	61	23	-	-	-	-	-	-	-	-	-	-
	14 DAI	196	190	84	72	41	266	173	93	69	55	-	-	-	-	-	-	-	-	-	-
	21 DAI	434	275	148	74	43	372	229	135	73	60	-	-	-	-	-	-	-	-	-	-
	28 DAI	403	269	124	77	54	404	326	182	123	65	-	-	-	-	-	-	-	-	-	-
Sub total (sheep)		***2361***	***1591***	***886***	***536***	***316***	***2454***	***1801***	***830***	***492***	***336***	-	-	-	-	-	-	-	-	-	-
Goat		Ivermectin : 0 mg/kg					Ivermectin at 0.4 mg/kg														
	Before	643	528	366	209	118	647	590	351	292	117	-	-	-	-	-	-	-	-	-	-
	2 DAI	493	380	159	108	69	502	446	170	137	62	-	-	-	-	-	-	-	-	-	-
	7 DAI	629	524	381	163	118	625	493	297	79	79	-	-	-	-	-	-	-	-	-	-
	14 DAI	547	365	35	89	64	515	297	128	68	68	-	-	-	-	-	-	-	-	-	-
	21 DAI	544	354	145	123	101	514	266	100	86	63	-	-	-	-	-	-	-	-	-	-
	28 DAI	384	258	109	65	44	372	209	58	55	34	-	-	-	-	-	-	-	-	-	-
Sub total (goat)		***3240***	***2409***	***1195***	***757***	***514***	***3175***	***2301***	***1104***	***717***	***423***	-	-	-	-	-	-	-	-	-	-
Pig		Ivermectin : 0 mg/kg					Ivermectin at 0.3 mg/kg					Ivermectin at 0.6 mg/kg					Ivermectin at 0.9 mg/kg				
	Before	359	165	70	54	45	301	195	90	47		373	191	106	76	58	325	171	82	61	37
	2 DAI	238	80	0	0	0	300	151	34	81	11	223	101	18	17	07	341	125	13	13	03
	7 DAI	371	284	223	211	80	292	198	67	51	29	343	198	25	17	08	333	258	36	27	09
	14 DAI	508	268	158	95	64	474	268	166	86	48	442	213	118	81	77	583	412	162	100	63
	21 DAI	234	130	52	25	20	233	98	17	13	05	267	181	84	52	45	199	137	50	36	48
	28 DAI	145	81	18	05	04	178	72	11	07	07	230	169	78	45	42	171	146	59	46	41
Subtotal (pig)		***1855***	***1008***	***521***	***390***	***213***	***1778***	***982***	***385***	***285***	***100***	***1878***	***1053***	***429***	***288***	***237***	***1952***	***1249***	***402***	***283***	***201***
**General total**		**7456**	**5008**	**2602**	**1683**	**1043**	**7407**	**5084**	**2319**	**1494**	**859**	**1878**	**1053**	**429**	**288**	**237**	**1952**	**1249**	**402**	**283**	**201**

Legend: DAI: Day after injection; T1: number of mosquitoes exposed at the first exposition; G1: number of mosquitoes that fed at T1; T2: number of mosquitoes exposed at the second blood-feeding; G2: number of mosquitoes that fed at T2; Diss: number of mosquitoes dissected for fecundity characterization.
