# Supplementary material for: Impact of blood meals taken on ivermectin-treated livestock on survival and egg production of the malaria vector Anopheles coluzzii under laboratory conditions
Source: PLoS One. 2024 Aug 15;19(8):e0308293. doi: 10.1371/journal.pone.0308293 (PMC11326554; doi:10.1371/journal.pone.0308293)
Supplement: S1 File — (PDF) [file pone.0308293.s002.pdf]

Supporting S1 File.

Preliminary study: efficacy of blood meal taken on treated pigs with therapeutic dose of ivermectin, on the survival of *Anopheles coluzzii*

The methodology was the same than the main study. Six (6) pigs were used in the activity (3 control and 3 injected subcutaneously with ivermectin at dose of 0.3 mg/ kg body weight.

A total of 4,155 females were exposed to pigs and 2,170 took blood meal, that is a global engorged rate of 52.27%.

Survival of An. coluzzii fed on pigs treated with therapeutic dose of ivermectin

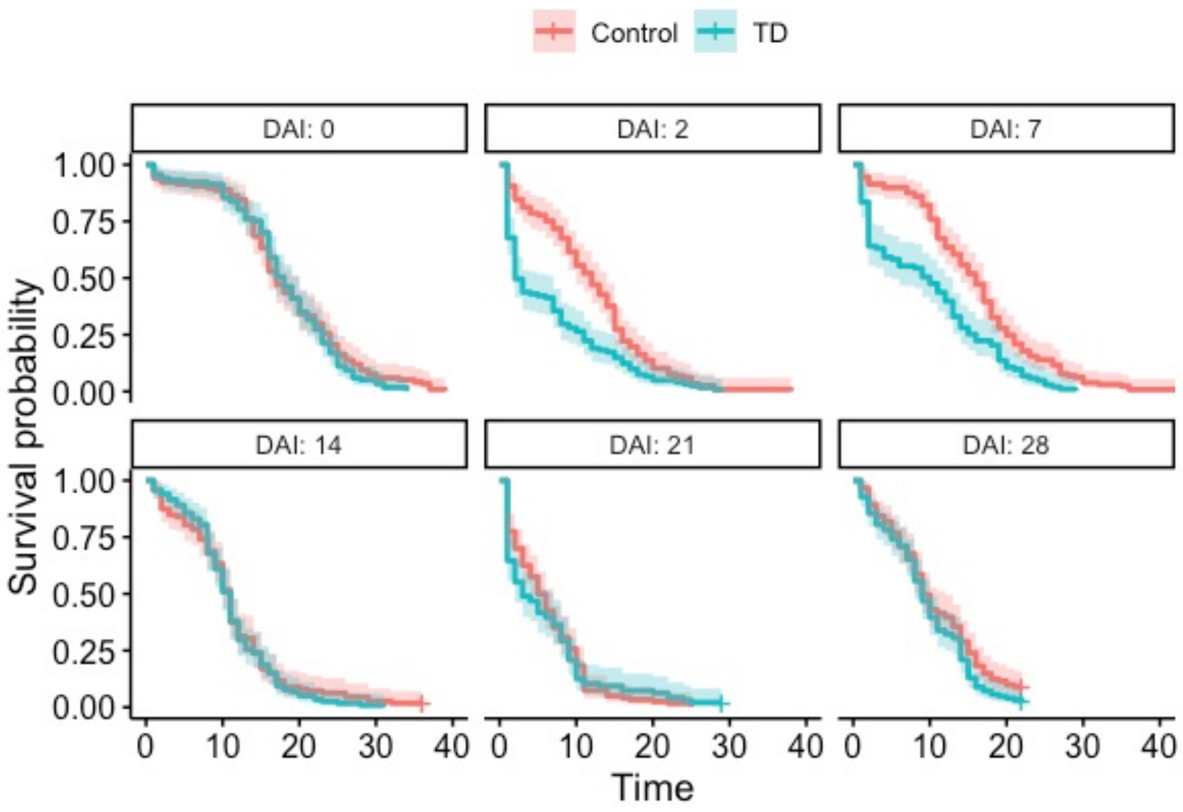

Figure 1. Kaplan-Meier curves showing the survival of mosquitoes fed on pigs from the control and therapeutic dose of ivermectin (TD = 0.3mg/kg) before treatment (DAI = 0) at different Days After Injection (DAI: 2, 7, 14, 21 and 28 days after injection of ivermectin dose.

Before the injection of ivermectin to the pigs during the first experiment, there was no significant effect to individual animal on the anopheles' survival ($X^2_5 = 6.51$; $P = 0.26$). After the injection of a recommended dose of ivemerctin to pigs, the analysis shows the significant difference of survival due to the effect of the delay post injection ($X^2_5 = 273.83$; $P < 0.001$), of the ivermectin treatment ($X^2_5 = 5.59$; $P = 0.02$), and of their interaction ($X^2_5 = 26.63$; $P < 0.001$) The ivermectin treatment decreased anopheles' survival on day 2 (HR = 1.89, IC [1.43 – 2.50], $P < 0.001$) and day 7 (HR = 1.92, IC [1.44 – 2.56], $P < 0.001$).

At 2 DAI, the median survival time of *Anopheles* was 2 days (IC= [2 – 7] days) for the treated group and 12 days (IC= [10 – 14] days) for the control group, representing a decrease of survival of 83.33 % in the treated group. At 7 DAI, the median survival time of *Anopheles* was 10 days (IC= [5 – 13] days) for the treated group whereas it was 16 days (IC= [14 – 17] days) for the control group, representing a decrease of 37.5%.
