# Supplementary material for: Impact of blood meals taken on ivermectin-treated livestock on survival and egg production of the malaria vector Anopheles coluzzii under laboratory conditions
Source: PLoS One. 2024 Aug 15;19(8):e0308293. doi: 10.1371/journal.pone.0308293 (PMC11326554; doi:10.1371/journal.pone.0308293)
Supplement: S2 File — (PDF) [file pone.0308293.s003.pdf]

<u>Supporting S2 file : Gravidity rate and fecundity in females *Anopheles coluzzii* fed on pig</u>

Before the injection of IVM, gravidity rates of *An. coluzzii* females were different for control and treated groups of pigs (Figure 1). There was a significant lower gravidity rate for females fed on the group of pigs to be treated with the therapeutic dose (OR = 4.00, IC [1.93 – 156], *P* = 0.02) and the double dose group (OR = 4, IC [1.23 – 11], *P* = 0.01) compared to those fed on the control one (Figure 1).

The only significant difference in gravidity rate was observed between *Anopheles coluzzii* females fed on control and treated pigs 7 DAI after injection of the therapeutic dose of ivermectin (OR = 7, IC [1.93 – 22], *P* = 0.004).

Also, the treatment with IVM did not show any effect on *An. coluzzii fecundity,* regardless the treatment dose or the DAI (Figure 2).

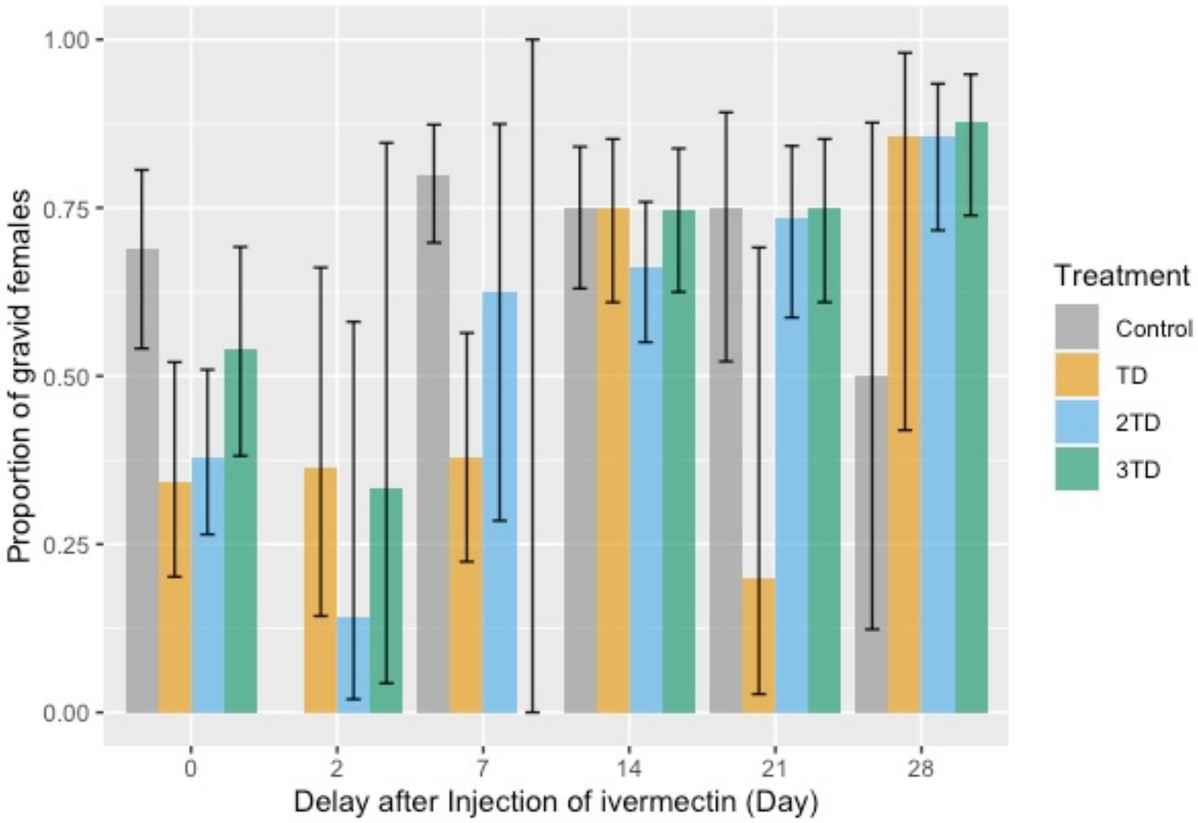

Figure 1. Proportion of gravid females of Anopheles coluzzii fed on control or treated pigs with different dose of ivermectin (TD: Therzpeutic dose = 0.3 mg/kg; 2TD = 2-fold therapeutic dose; 3TD = 3-fold therapeutic dose). The error bars correspond to the Standard error.

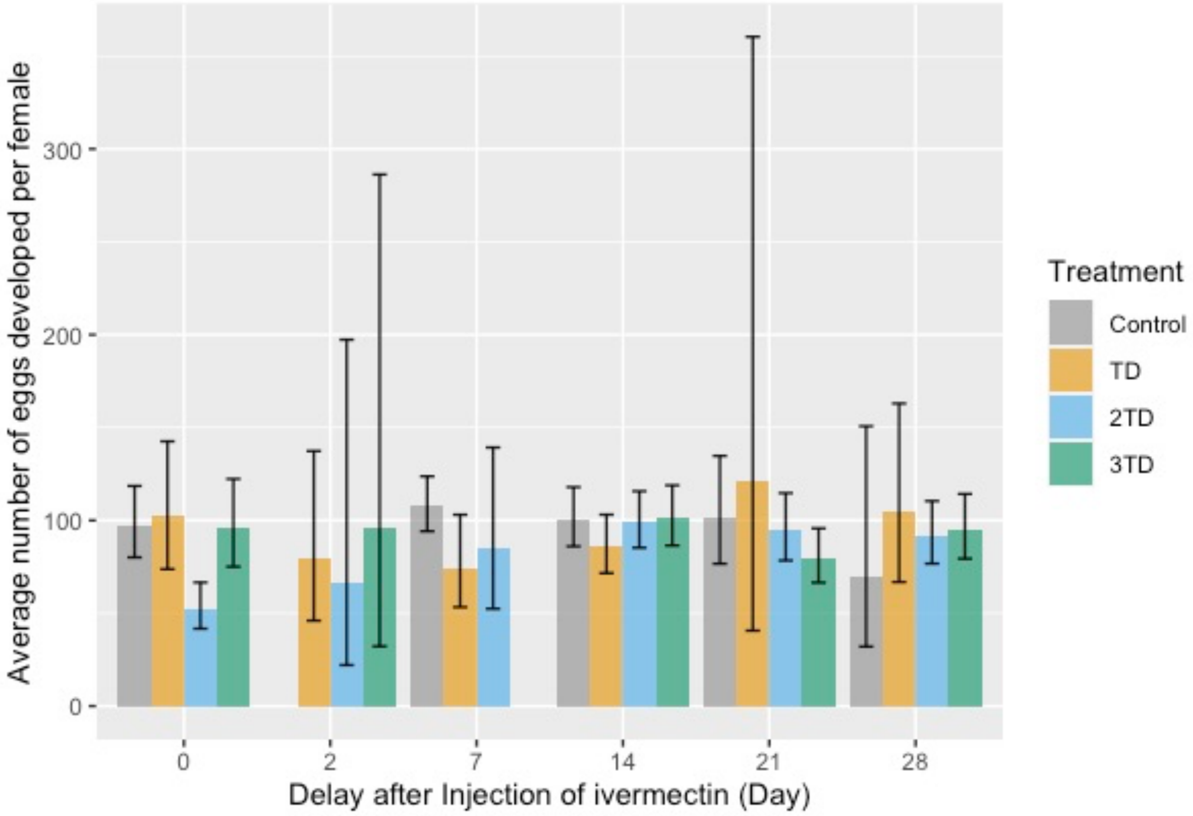

Figure 2. Average number of eggs developed by gravid females of Anopheles coluzzii fed on control or treated pigs with different dose of ivermectin (TD: Therzpeutic dose = 0.3 mg/kg; 2TD = 2-fold therapeutic dose; 3TD = 3-fold therapeutic dose). The error bars correspond to the Standar error.
