# Supplementary material for: Impact of blood meals taken on ivermectin-treated livestock on survival and egg production of the malaria vector Anopheles coluzzii under laboratory conditions
Source: PLoS One. 2024 Aug 15;19(8):e0308293. doi: 10.1371/journal.pone.0308293 (PMC11326554; doi:10.1371/journal.pone.0308293)
Supplement: Attachment — Submitted filename: Response to Reviewers.pdf [file pone.0308293.s008.pdf]

PONE-D- 23-39281

Impact of blood meals taken on ivermectin-treated livestock on survival and fecundity of the malaria vector Anopheles coluzzii under laboratory conditions

Responses to reviewers

Journal requirements:

1. When submitting your revision, we need you to address these additional requirements.

Please ensure that your manuscript meets PLOS ONE's style requirements, including those for file naming. The PLOS ONE style templates can be found at https://journals.plos.org/plosone/s/file?id=wjVg/PLOSOne_formatting_sample_main_body.pdf and https://journals.plos.org/plosone/s/file?id=ba62/PLOSOne_formatting_sample_title_authors_affiliations.pdf.

Response: *These requesting has been taken account.*

2. In your Methods section, please provide additional information regarding the permits you obtained for the work. Please ensure you have included the full name of the authority that approved the field site access and, if no permits were required, a brief statement explaining why.

Response*: Information about the permits obtained for the work has been added to the Materials and Methods' section of the revised* ***manuscript (lines 118–121)****.*

3. We suggest you thoroughly copyedit your manuscript for language usage, spelling, and grammar. If you do not know anyone who can help you do this, you may wish to consider employing a professional scientific editing service.

Whilst you may use any professional scientific editing service of your choice, PLOS has partnered with both American Journal Experts (AJE) and Editage to provide discounted services to PLOS authors. Both organizations have experience helping authors meet PLOS guidelines and can provide language editing, translation, manuscript formatting, and figure formatting to ensure your manuscript meets our submission guidelines. To take advantage of our partnership with AJE, visit the AJE website (http://learn.aje.com/plos/) for a 15% discount off AJE services. To take advantage of our partnership with Editage, visit the Editage website (www.editage.com) and enter referral code PLOSEDIT for a 15% discount off Editage services. If the PLOS editorial team finds any language issues in text that either AJE or Editage has edited, the service provider will re-edit the text for free.

Upon resubmission, please provide the following:

The name of the colleague or the details of the professional service that edited your manuscript

A copy of your manuscript showing your changes by either highlighting them or using track changes (uploaded as a *supporting information* file)

A clean copy of the edited manuscript (uploaded as the new *manuscript* file)".

***Response**: These requesting has been taken account. The co-authors edited the manuscript.*

4. We note that the grant information you provided in the 'Funding Information' and 'Financial Disclosure' sections do not match.

When you resubmit, please ensure that you provide the correct grant numbers for the awards you received for your study in the 'Funding Information' section.

***Response**: This has been corrected in the revised manuscript **(lines 492-495)**.*

5. Your ethics statement should only appear in the Methods section of your manuscript. If your ethics statement is written in any section besides the Methods, please move it to the Methods section and delete it from any other section. Please ensure that your ethics statement is included in your manuscript, as the ethics statement entered into the online submission form will not be published alongside your manuscript.

***Response**: The ethics statement has been moved to the Materials and Methods section as suggested (lines 118–121).*

6. Please include captions for your Supporting Information files at the end of your manuscript, and update any in-text citations to match accordingly. Please see our Supporting Information guidelines for more information: http://journals.plos.org/plosone/s/supporting-information.

***Response:** The changes have been made in the Supporting Caption on **lines 476-481**.*

Review Comments to the Author

Reviewer #1:

Pooda and Colls investigated the effects of ivermectin treatments as an ectocide in three livestock species (Sheep, goats and pigs) on the survival and reproduction of the malaria vector Anopheles coluzzii. Different domestic animal species have been used since the pharmacokinetics of Ivermectin may vary between vertebrate hosts. This strategy could be used to complement current control tools such as LLINs and IRS to target exophagic, exophilic and zoophagic vectors. The injectable veterinary ivermectin formulation at the species-specific doses caused a significant decrease in mosquito survival for up to 7 days after injection. The number of gravid females Anopheles that survived after feeding on treated animals was also reduced, as well as the number of mature eggs in the ovaries. However, due to the short-term efficacy of single-dose treatments, repeated treatments and potentially increased dosages would be required to span the transmission season.

The methodology seems to be appropriate, and the results obtained support the conclusions realised by the authors. However, some issues and concerns should be addressed.

Response: *We thank sincerely Dr Marcos Sterkel for the reading and analyzing our paper. We appreciated greatly your observations made about this work.*

1. **The authors considered the proportion of females carrying eggs (gravidity rate) and the number of mature eggs in the ovaries as proxies of mosquito fecundity. It is not the optimal way to assess the effect of a drug on reproductive fitness. The presence of a drug may delay ovaries and egg development without affecting the final reproductive output. A better and more direct way to assess reproductive fitness would be to allow the treated females to complete the reproductive cycle, lay the eggs, and count the number of eggs laid by each female and their hatching rate. The final number of F1 per female is the better way to quantify the effect of a drug on reproductive fitness.**

Response: *We agree that using only the number of mosquito females carrying eggs and the number of mature eggs per female gives only part of the answer on the impact of ivermectin on the overall reproductive fitness of* Anopheles coluzzii. *We are however confident that we are not observing a delay in ovaries and egg development and that the number of eggs we counted by gravid female represents the final output in terms of the maximum number of eggs that a female could carry, since we let the female to develop eggs for 4 days post blood-meal, and since during dissection, all the eggs observed and counted under a binocular were mature (Christopher stage 5). Moreover, no remaining, undigested blood, has been observed. We are however aware about the fact that these counts (% gravids and number of eggs per female) represent only a "potential" and that the actual number of laid eggs and, moreover, the number of larvae that develop from these eggs illustrate better the actual reproductive fitness. Although other authors used as well the number of eggs counted through dissection as a fecundity index (Mekuriaw et al. Malar J (2019) 18:357 https://doi.org/10.1186/s12936-019-2988-3), we took into consideration the reviewer's remark and modified several sentences accordingly throughout the text, including the manuscript title (fecundity was replaced by eggs production). We directly addressed this issue in the materials and methods section*, ***lines 207-210****, by acknowledging the fact that we present a proxy of the fecundity expressing a potential only.*

“The proportion of females carrying developed eggs (gravidity rate) and the number of mature eggs (i.e., those that reached Christopher stage V of ovarian development) [39] are proxies representing important parameters of the mosquitoes reproductive potential.”

2. **Which is the insecticide resistance status of the An. coluzzii colony used? Please add this information, if known.**

Response: *The colony we used during our experiments in 2017 and 2018 was the same as in the study from Pooda et al. 2015. Like stated in this article, the colony was repeatedly replenished with wild mosquitoes from the village of Bama, where founder individuals were collected as well and where the mutated kdr allele prevalence is very high. We therefore assume that we also dealt with mosquito batches displaying the same proportion of pyrethroid resistant mosquitoes than previously reported, which was 30-40%. This information is now added in the manuscript* ***lines 123-134.***

“A colony of one of the major vectors of *Plasmodium*, *An. coluzzii,* was used in this study. The colony was established in year 2008 from 200 wild blood-fed females captured inside houses using a mouth aspirator at the Kou Valley (11 ° 23’14 ” N, 4 ° 24’42 ” W) near Bobo-Dioulasso, South-Western Burkina Faso, was used in this study. It is the same than the one used for the study by Pooda et al. (2015), with a proportion of 30-40% mosquitoes carrying the *kdr*-resistant allele conferring resistance to pyrethroids. It was repeatedly replenished with F1 from wild-caught mosquito females collected in the same area. This *Anopheles* species is one of the major vectors of *Plasmodium* parasites in Burkina Faso [33]. The species composition of the colony, its resistance to insecticides status, and potential contamination by other species or strains was routinely checked using PCR as previously described [34] »

3. **Lines 235-236: The rate of blood-fed mosquitoes was, respectively, 71.52 (±4.88) %, 71.94 (±3.15) % and 57.46 233 (±2.55) % on sheep, goat and pig at the first blood meal (Figure 1).**

   Were these values calculated using both control and Ivermectin-treated animals? Please clarify this issue.

Response: *The values presented here were calculated with the overall data considering mosquitoes fed on control and treated animals. This is because there were no significant differences between treatments. Details of the number of mosquitoes fully engorged for each group of animals and at all considered time points are given in the Supplementary Table S1. The reviewer’s comment has been taken into account and the reported sentence has been completed as follows****, lines 248-255:***

“There was no effect of the treatment of ivermectin on the rate of mosquitoes blood-fed on sheep (χ^2_1=0.0867, P = 0.77), goats (χ^2_1=0.1071, P = 0.74) and on pigs (χ^2_3=2.5833, P = 0.46), with no significant difference between mosquitoes fed on corresponding treated and control animals (**S1 Table**). All samples taken together, the rate of blood-fed mosquitoes during the first blood meal was, respectively, 71.52 (±4.88) %, 71.94 (±3.15) % and 57.46 (±2.55) % on sheep, goat and pig at the first blood meal (**Figure 1**), and was, for the second blood meal, 59.57 (±4.55) %, 58.26 (±5.53) %, and 69.46 (±2.74) %. The sample size for each mosquito group is given in the supporting **S1 Table**”.

4. Figure 1: Change DT for TD in the X-axis information.

Response: *This has been done, see the Figure 1*

What's the meaning of ten in the X-axis legend? This figure is difficult to interpret and contains some mistakes. I guess the pink columns are the insects fed on control animals, blue are mosquitoes fed on therapeutical dose-treated animals (TD), while green and violet colours are 2TD and 3DT in mosquitoes fed on pigs. Please clarify this and accommodate the order of columns in panel C according to panels A and B. Add ¨3TD¨ at figure legend.

Response: *We thank the reviewer for his remarks to improving the quality of our figure. However, we are quite puzzled because they do not seem to fit if we consider the Figure 1 that we believe we submitted together with our manuscript (see below). We are confused and we very much apologize because the mistake must come from our side with a preliminary instead of final Figure 1 submitted. We hope that the reviewer will find the actual Figure 1 below being suitable for illustrating our results about the proportion of Anopheles females that fed on hosts of different species treated with different ivermectin doses. As for answering to the question "***What's the meaning of ten in the X-axis legend?",** there is no more "10" in the X-axis legend.

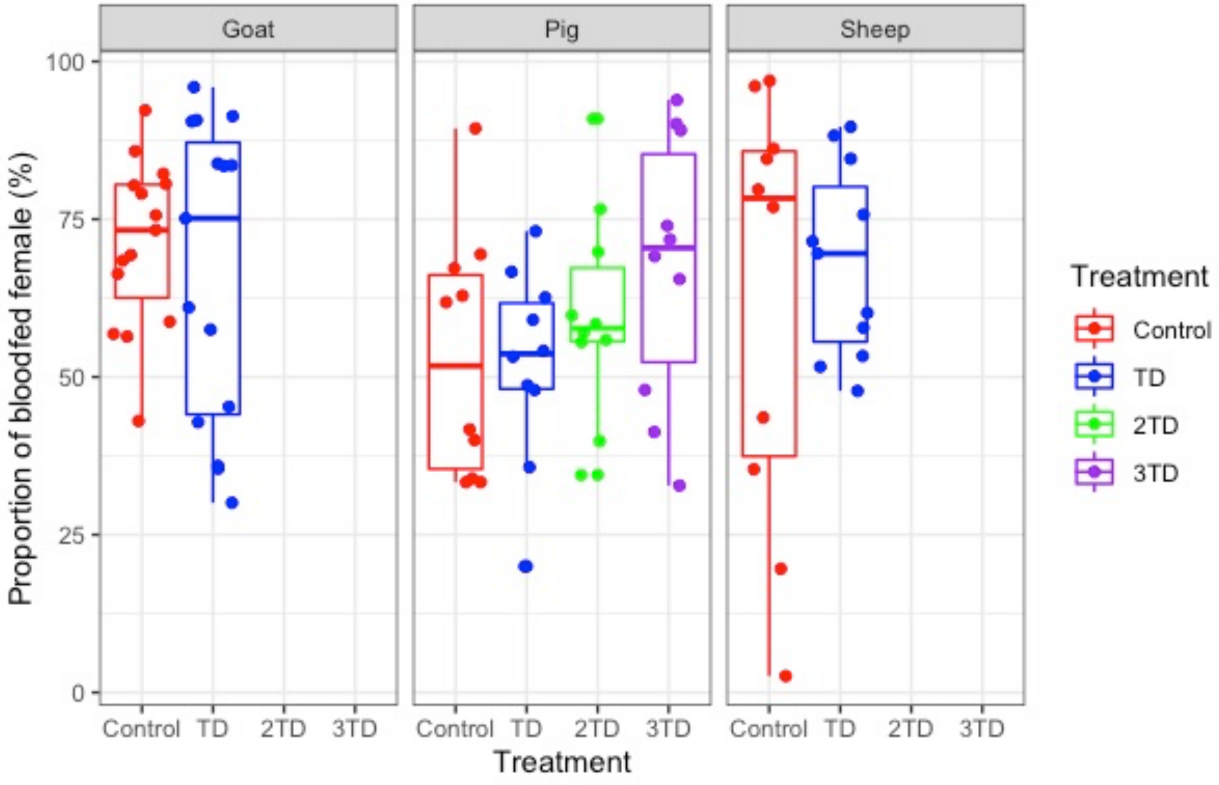

Figure 1: Rate of blood-fed Anopheles coluzzii according to host species and ivermectin treatment.

5. Line 255-256: The doubled and tripled therapeutic doses used to treat pigs induced as well a significant decrease in mosquito mortality rates

Should be ¨ a significant increase

Response: *This has been corrected in the manuscript. This reads now at* ***lines 298 - 306****.*

6. A. Figure 2: There seems not to be differences in survival in IVM-treated and control goats on day 2 DPI. Please check it.

Response: *Thank you for your observations. For this specific experimental point at 2DAI, the statistical analyses showed a significant decrease in Anopheles survival between IVM-treated and control goats (HR = 1.46, IC [1.09 – 1.95]; P= 0.01). The effect is not graphically obvious due to the duration of the follow up per se (until all mosquitoes died) but also, an unexpected low survival rate in the control arm occurred at this DAI. However, this result is statistically supported* ***(lines: 281-286)****. The median survival at 2 DAI was 9 days for the control mosquitoes (8, 8, and 9 days for the three goats, respectively) and 7 days for mosquitoes that fed on treated goats (7, 6, and 7 days for the three IVM-treated goats, respectively).*

6. B. Besides, the mortality in pigs is higher for TD than for 2TD on day 2 DPI. Please also check this issue.

Response*: To better visualize mortality data we obtained when exposing mosquitoes to control and treated pigs 2 days after injection of ivermectin, we plotted below the survival curves for this DAI and for the different treatments. We can notice the variability of the toxic effect generated by blood feeding on the pigs P3 and P6 from the 2TD, and also the TD treatments. Such variability is directly linkable to the variability observed in ivermectin concentration values (see table 2 below).*

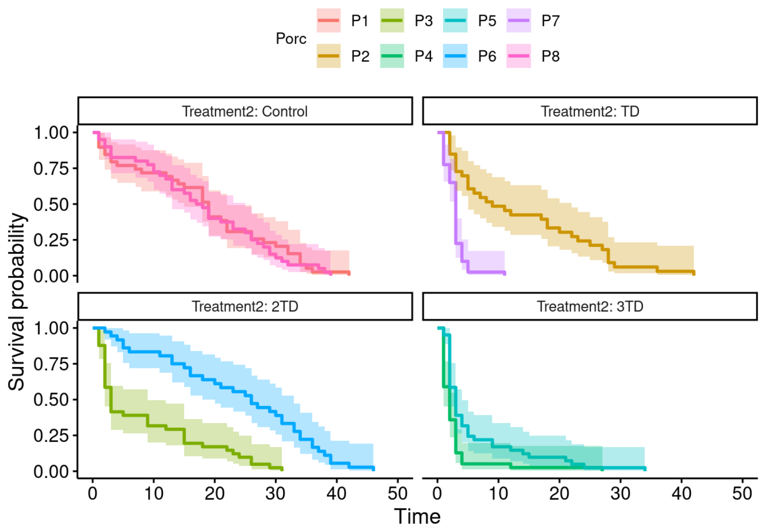

Figure 2. Survival curves for mosquitoes from the same lot fed on control (P1 and P8) and treated pigs 2 DAI after treatments. Ivermectin treatments were injected at the therapeutic dose (TD, P2 and P7), the double (2TD, P3 and P6) and the triple (3TD, P4 and P5) therapeutic dose.

The pharmacokinetic/pharmacodynamic of ivermectin varies greatly among species, and, considering the same species, it varies also greatly among individuals, depending mostly on the treated animal's weight, physiology (in particular fat tissue percentage) and the injection act per se, which is subject to variations (in precision and speed) from an animal to another because animals' tremors, movements, and because human variability from an injection to another as well.

Pharmacokinetics variability among the 2 animals of the 2TD treatment (for the reasons mentioned above) might be at the origin of the results observed between TD and 2TD that the reviewer mentioned, with one from both displaying an ivermectin concentration that is as low as the one observed at 14 DAI for the TD treatment (6-7 ng/ml) where variability between animals is low and where this concentration is not associated with significant mortality in mosquito batches fed on these treated pigs. For the same species, inter-individual PK variability is highlighted in table 2 and was already discussed in our manuscript, ***lines 399-401****. This is now mentioned in the results section as well,* ***lines 355 – 356:*** "For pigs in particular, a great inter-individual host variability in ivermectin plasma concentration can be noticed." *This issue would be solved using more pigs per treatment. However, already published data considered 2 pigs only as well in their protocol* (Pasay *et al. Parasites Vectors (2019) 12:124* https://doi.org/10.1186/s13071-019-3392-0).

6.C. Add the time units in the X-axis (days?)

"Days after blood meal" as the time units have been added to the Figure as requested.

7. Line 282-283: From day 14 post-treatment and onwards, there was no significant difference between groups whatever the host species considered (Figure 1).

Should be ¨ Figure 2¨

Response: *Thank you very much. This has been corrected in the manuscript at now* ***lines 296-297****.*

8. Figures 3 and 4: Please, add asterisks to the statically significant differences between control and IVM-treated animals.

Response: *Asterisks have been added to the statically significant differences between control and IVM-treated animals as suggested.*

9.A. There is no correlation in the mean plasma concentrations of ivermectin (ng/mL) in the treated-sheep, goats and pigs (table II) with the mortality observed in Figure 2 and the number of eggs in Figure 4. Can the authors explain this?

9.B. For example, in pigs, only the 3TD caused increased mortality on day 14 DPI, but the concentration is as high as 2TD, which did not increase mortality on that day.

9.C. Besides, 2TD on pigs doubles the concentration in goats at 2 DPI and in sheep at 7 DPI, and both increased mosquitoes' mortality. A deeper discussion about the lack of correlation in plasma IVM concentrations and the phenotypes observed associated with reduced survival and reproductive fitness is needed. The lack of correlation makes the interpretation of the results very difficult.

9.A and 9.C. Inter-species variability in PK/PD:

Response: *There is evident great discrepancy in plasma levels between species, and not only because the administered doses are different (see Table 2). This has been already reported (Alvinerie et al., 1998; Veterinary Research, 1998, 29: 113-18*) *and is highly explained by the fact that plasma ivermectin concentrations of a given species result from different parameters that are species-dependent: the volume of distribution of the molecule, the different body compartments where the molecule is actually distributed, the fat body percentage as ivermectin is highly hydrophobic, and, lastly, the metabolism of the considered species. All these parameters actually determine quantitatively and qualitatively the molecule's distribution in the different body compartments, including the skin's blood capillaries where the mosquitoes bite, and herein its bio-availability to mosquitoes and mosquitocidal efficacy. Given the species-dependent nature of each of these parameters, the relationship between ivermectin plasma pharmacokinetics and its actual bioavailability for mosquitoes obviously differs from a species to another. In other words, the effects of ivermectin on mosquitoes' mortality should not be extrapolated nor compared from a species to another based on plasma concentrations only. Correlations between efficacy (at reducing survival and/or reproductive fitness) and plasma ivermectin concentrations are only species specific. For proper projection of field effectiveness of treating peridomestic animals using ivermectin against malaria, species specific PK/PD relationships should therefore be drawn. To illustrate this in the frame of our experiment, we calculated the 7-day LC50 value for each species using PK and survival data. Computation of the 7-day LC50 was possible using the drc R package (Ritz et al. 2015, Plos One, 10 (12); doi:10.1371/journal.pone.0146021). However, for sheep and goats, the model fitting had to be constrained on some parameters (Lower Limit, Upper Limit) which implies that results are not robust, especially for sheep since ivermectin plasma concentrations were available only for a single time point which preclude any robust PK/PD analysis. For pigs, fitting is allowed without constraints and models outputs can therefore be safely taken into consideration. The fits to the data are presented below for goats and pigs, and models outputs as well.*

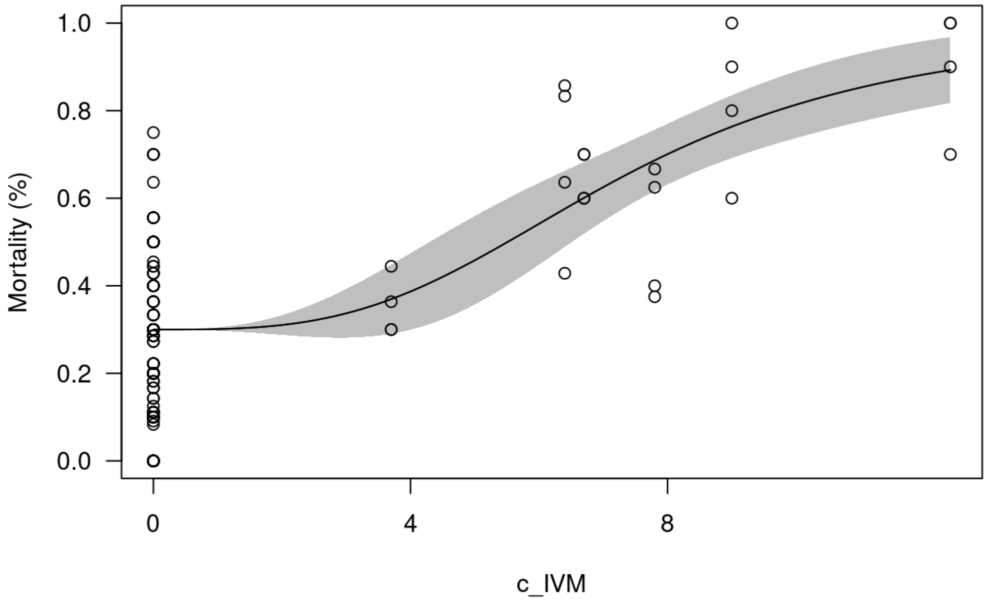

Figure 1. Seven-days cumulated mosquito mortalities in function of ivermectin concentrations (in ng/ml plasma) measured in goats.

The parameters estimate for the corresponding drc model (see Figure 1) are given below (slope, lower and upper limits were arbitrarily fixed, Slope = -1, Lower Limit = 0, Upper Limit = 1). 7-Days LC50, se and the p—value are highlighted in yellow.

```
                  Estimate Std. Error t-value   p-value
Slope:(Intercept) -3.23886    0.81079 -3.9947 6.478e-05 ***
ED50:(Intercept)   7.31143    0.48461 15.0874 < 2.2e-16 ***
---
Signif. codes:  0 '***' 0.001 '**' 0.01 '*' 0.05 '.' 0.1 ' ' 1
```

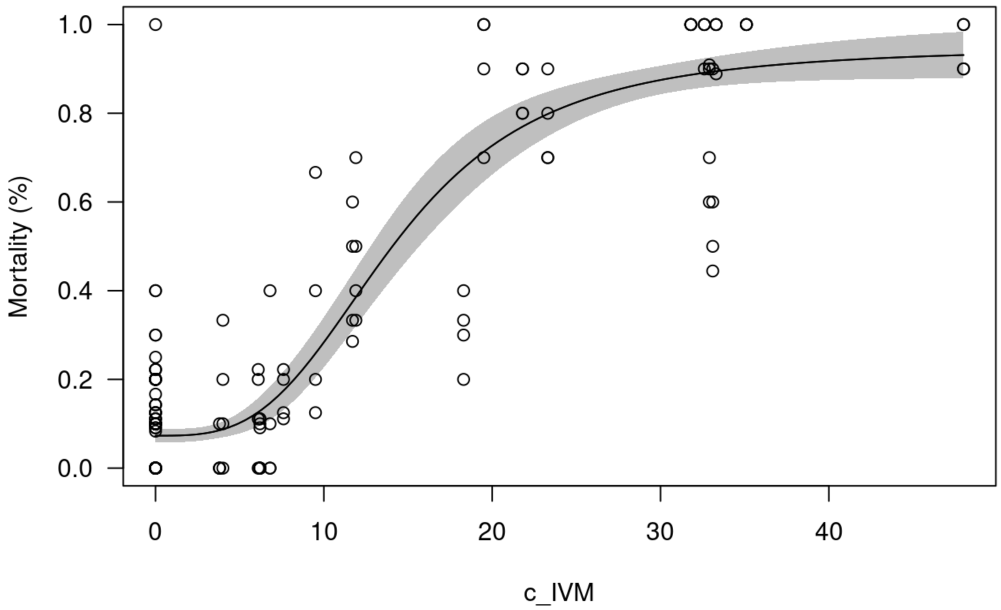

Figure 2. Seven-days cumulated mosquito mortalities in function of ivermectin concentrations (in ng/ml plasma) measured in pigs. All data from the different treatments are combined.

Model outputs are given below. The 7-Days LC50, se and p-value are highlighted in yellow.

```
Parameter estimates:

                          Estimate Std. Error t-value   p-value
Slope:(Intercept)       -3.2209585  0.4358817 -7.3895 1.474e-13 ***
Lower Limit:(Intercept)  0.0731089  0.0078757  9.2829 < 2.2e-16 ***
Upper Limit:(Intercept)  0.9485856  0.0369034 25.7046 < 2.2e-16 ***
ED50:(Intercept)        14.2891676  1.0123208 14.1153 < 2.2e-16 ***

---
```

These data illustrate that 7-days LC50 are different between goats and pigs. Although preliminary, these new data are interesting and are in line with our discussion about PK/PD differences among species. However, we chose not to present them in our corrected manuscript due to the lack of robustness of the modeling and corresponding outputs for goats.

9.B. Inter-individual PK/PD variability in the same species

Response*: This question relates to the same remark as in the question 6.B. and the same answer applies, concerning the great inter-individual variability in pigs plasma concentrations and this limitation of our study that we acknowledge, as did authors of the following study where 2 pigs were used as well (*Pasay *et al. Parasites Vectors (2019) 12:124* https://doi.org/10.1186/s13071-019-3392-0*).*

Reviewer #2: PONE-D- 23-39281

Impact of blood meals taken on ivermectin-treated livestock on survival and fecundity of the malaria vector Anopheles coluzzii under laboratory conditions

This review is by Carlos Chaccour from ISGlobal, Barcelona Institute for Global Health. I have a personal open peer-review policy as the current single-blinded system is riddled with vices.

This manuscript reports the results of an experiment conducted in Burkina Faso in which pigs, sheep and goats were treated with different doses of ivermectin and then An. coluzzii mosquitoes were fed on them at different times after treatment. The authors also collected some PK data. The results are discussed on the context of a potential One Health approach to malaria control.

Albeit some minor mistakes, the manuscript is well written. The methods are appropriate for the objectives and the conclusion is supported by the result. I provide here comments for the author`s consideration.

Response: *We thank sincerely Dr Carlos Chaccour for its precious observations and comments.*

Introduction

1. **Consider mentioning early exit as another mosquito behavior contributing to residual transmission. This can be induced by the repellent properties of indoor insecticides or occur even in their absence.**

Response*: This specific behaviour has been introduced in the manuscript, and the corresponding sentence modified accordingly, see* ***lines 75-79***

« The main challenges with LLINs and IRS strategies are the persistence of residual transmission of Plasmodium due to mosquito populations resistant to insecticides (*i.e.* metabolic and target site mutations), or displaying behaviors that limit or avoid the contact with the molecules (*i.e.* feeding on livestock, biting and resting outdoors, early or late aggressive behavior, early exit from houses to evade indoor insecticide exposure, etc.) »

2. **There is no mention of two key concepts: zooprophylaxis vs zoopotentiation.**

Response*: These concepts are now integrated in our text****, lines 91-96***.

« These animals that usually live near human populations also represent an alternative blood source for malaria vectors that enables their reproduction and survival, hence sustaining their role in *Plasmodium* transmission and leaning toward zoopotentiation [22,23]. Therefore, using ivermectin for treating a large panel of peridomestic animals would represent an endectocide-based zooprophylactic approach, in the frame of the One-Health concept, which would virtuously intricate human's and animal's health [24] ».

The term zooprophylaxis has been added in the discussion section as well, in the following sentence ***lines 364 - 366****:* "However, this feeding behavior may provide an opportunity for zooprophylaxis and to controlling these malaria vectors using the insecticide-treated livestock (ITL) strategy »

3. **Methods**
   3.A. Please mention the colony`s insecticide resistant status. This is important given the potential cross-metabolic resistance with pyrethroids as they share the same CYP as ivermectin.

Response: *The colony we used during our experiments in 2017 and 2018 was the same as in the study from Pooda et al. 2015. Like stated in this article, the colony was repeatedly replenished with wild mosquitoes from the village of Bama, where founder individuals were collected as well and where the mutated kdr allele prevalence is very high. We therefore assume that we also dealt with mosquito batches displaying the same proportion of pyrethroid resistant mosquitoes than previously reported, which was 30-40%. This information is now added in the manuscript* ***lines 123-131****.*

- "A colony of one of the major vectors of *Plasmodium*, *An. coluzzii,* was used in this study. The colony was established in year 2008 from 200 wild blood-fed females captured inside houses using a mouth aspirator at the Kou Valley (11 ° 23′14 ″ N, 4 ° 24′42 ″ W) near Bobo-Dioulasso, South-Western Burkina Faso, was used in this study. It is the same than the one used for the study by Pooda et al. (2015), with a proportion of 30-40% mosquitoes carrying the *kdr*-resistant allele conferring resistance to pyrethroids. It was repeatedly replenished with F1 from wild-caught mosquito females collected in the same area. This *Anopheles* species is one of the major vectors of *Plasmodium* parasites in Burkina Faso [33]. The species composition of the colony, its resistance to insecticides status, and potential contamination by other species or strains was routinely checked using PCR as previously described [34] »

Because this colony was not raised under pyrethroids pressure, we believe that metabolic resistance, induced by insecticides presence, actually vanished quickly and that the phenotypes we observe are not the consequence of cross resistance due metabolic mechanisms.

3.B. Please mention the calculated adipose vs lean weight of the animals.

Response: *Despite important search in the available literature, we failed at finding how to calculate adipose and lean weight of our animals. To our knowledge, such values are measured with a balance using bio-electrical impedance analysis, and this was not the type of balance available in our facilities. Except the weight before injection measured using an electrical balance, no other body parameter (size at the withers) has been taken into account to unable obtaining such parameters through calculation. Averaged fat percentages are given in the literature for the different species we used (Schumacher et al, Animals, 20222 (12), 1550; DOI: 10.3390/ani12121550). Values are variable, depending among other parameters, on the species, the race type, the metabolism, and the food intakes, in quality and quantity. This was not therefore generalizable to our experimental context.*

We understand that our reviewer would have liked to see mentioned the different fat contents vs lean weight among the different considered species to decipher about their respective influence on ivermectin PK and efficacy duration. Although without these concrete data, the different body composition and metabolism

among species and the related impacts on ivermectin distribution in the different body compartment are discussed in our manuscript. See ***lines 377-392.***

We would be more than happy to compute these parameters if the reviewer gives us more insights into how to proceed.

3.C. How was the random allocation of mosquito cups done? Were cups rotated in the insectary?

Response*: We added the complementary description on* ***lines 180-186****, as bellow:*

"All mosquitoes fed on the same animal were transferred in a large cage from which mosquitoes were individually aspirated using a mouth aspirator and sequentially put in the cups (cup 1 to 4 and then back again to cup 1) until cups were completed to 10 mosquitoes each. All cups were put in trays, and on a shelf, in the insectary. Each day, the cups were taken from the trays, observed for mosquito mortality and put back. To avoid confounding positional effect on mosquito mortality and fecundity phenotypes, trays were rotated from shelf to shelf, and cups inside the trays as well. All the cups were maintained in the same insectary".

4. **Figure 2 is pixelated, making reading it difficult. Additionally, the X axis seems compressed, giving the illusion that the curves are smooth rather than the usual K-M step by step drop. I recommend providing a higher quality image and perhaps even separating it in three different figures to ensure sufficient size. Consider also adding guiding marks to the reader such as the median survival in the control group.**

Response: *Thank you for your observation. The suggested modifications have been made to improve the readers' understanding. We did not endorse the recommendation about drawing a figure per species because we think that having all curves in the same panel gives the opportunity to compare treatments mosquitocidal efficacy in a quick glance.*

5. **Results**
   5.1. The increased gravity found at 28 DAI in goats does not seem to be statistically significant as the CI overlap in figure 3. If that is the case, I recommend, stating it in the text. The same for the decrease in fecundity reported at day 28 in sheep, or 7 DAI in goats, although in these cases p-values are provided in the text, it is worth it to double check the figure given that CI-overlap.

Response: *Thank you very much to the reviewer for raising this lack of statement concerning the lack of significance of increased gravidity at 28 DAI when colony females fed on treated goats. A statement has been added to the revised manuscript, and the corresponding sentence now reads* ***(lines 326-329)****:* "However, an unexpected 39.76% marginally significant increase in gravidity rate was observed in *An. coluzzii* that fed on treated-goats at 28 DAI compared to those fed on control animals (79.41% ± 6.93% vs 56.82% ± 7.46% in the control group, Figure 3, OR = 0.34, IC [0.123 – 0.949], P = 0.04)". *For the data reported at 28 DAI (sheep) or 7 DAI (goats), we double checked for significance and the provided associated p-values are indeed correct.*

5.2. Table II. The metric should be median and range given the samples come from only two animals.

The median ivermectin concentrations values and the ranges are now given in table 2.

5.3. Can the authors use the PK data to estimate the 7-day LC50?

***Response**: Computation of the 7-day LC50 was possible using the drc R package (Ritz et al. 2015, Plos One, 10 (12); doi:10.1371/journal.pone.0146021). However, for sheep and goats, the model fitting had to be constrained on some parameters (Lower Limit, Upper Limit) which implies that results are not robust, especially for sheep since ivermectin plasma concentrations were available only for a single time point which preclude any robust PK/PD analysis. For pigs, fitting is allowed without constraints and models outputs can therefore be safely taken into consideration. The fits to the data are presented below for goats and pigs, and models outputs as well.*

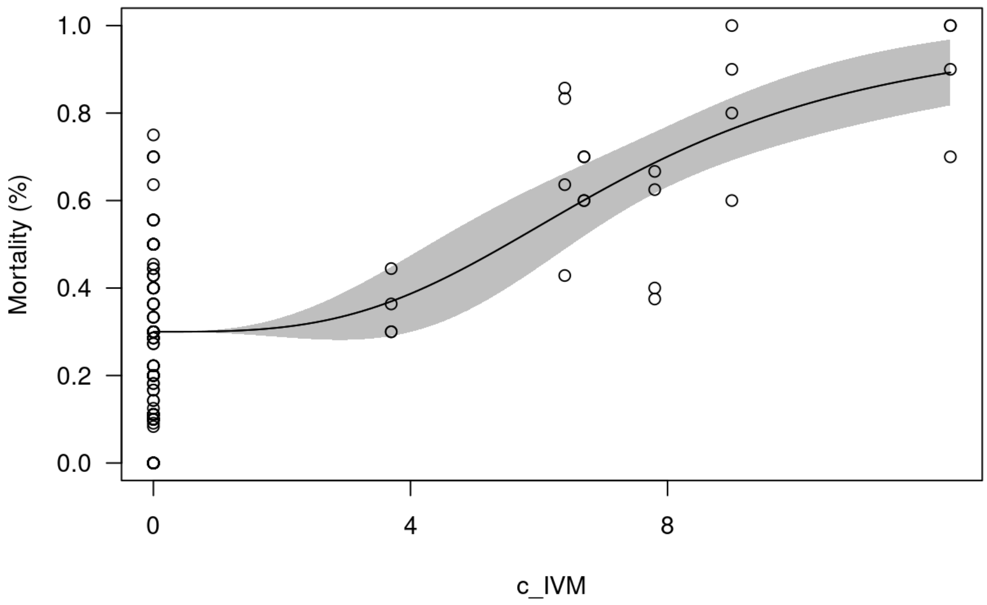

Figure 1. Seven-days cumulated mosquito mortalities in function of ivermectin concentrations (in ng/ml plasma) measured in goats.

The parameters estimate for the corresponding drc model (see Figure 1) are given below (slope, lower and upper limits were arbitrarily fixed, Slope = -1, Lower Limit = 0, Upper Limit = 1). 7-Days LC50, se and the p—value are highlighted in yellow.

```
Parameters estimate:

                  Estimate Std. Error t-value   p-value
Slope:(Intercept) -3.23886    0.81079 -3.9947 6.478e-05 ***
ED50:(Intercept)   7.31143    0.48461 15.0874 < 2.2e-16 ***
---

Signif. codes:  0 '***' 0.001 '**' 0.01 '*' 0.05 '.' 0.1 ' ' 1
```

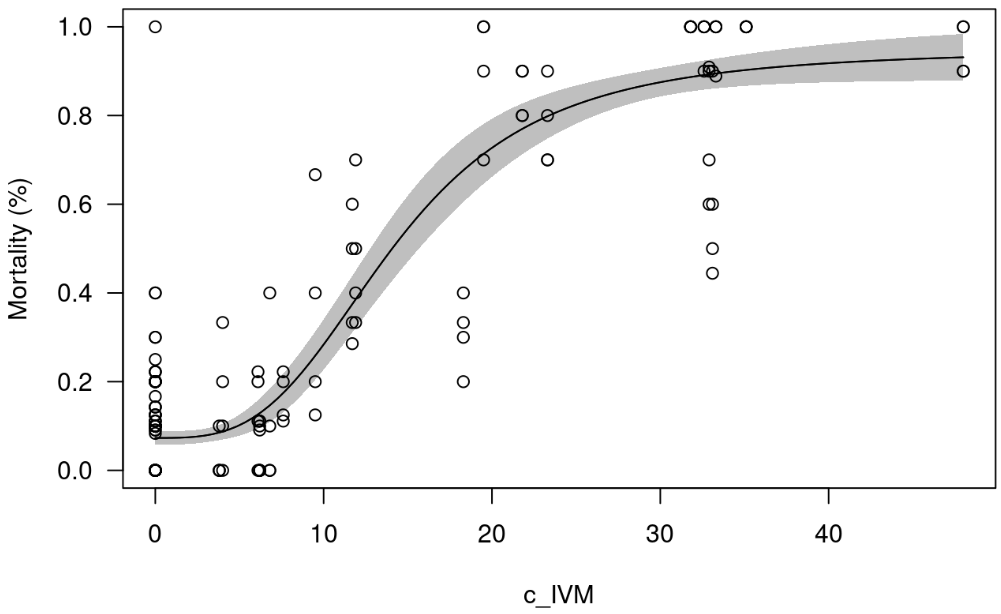

Figure 2. Seven-days cumulated mosquito mortalities in function of ivermectin concentrations (in ng/ml plasma) measured in pigs. All data from the different treatments are combined.

Model outputs are given below. The 7-Days LC50, se and p-value are highlighted in yellow.

```
Parameter estimates:

                          Estimate Std. Error t-value   p-value
Slope:(Intercept)       -3.2209585  0.4358817 -7.3895 1.474e-13 ***
Lower Limit:(Intercept)  0.0731089  0.0078757  9.2829 < 2.2e-16 ***
Upper Limit:(Intercept)  0.9485856  0.0369034 25.7046 < 2.2e-16 ***
ED50:(Intercept)        14.2891676  1.0123208 14.1153 < 2.2e-16 ***

---
```

These data illustrate that 7-days LC50 are different between goats and pigs. Although preliminary, these new data are interesting and are in line with our discussion about PK/PD differences among species. However, we chose not to present them in our corrected manuscript due to the lack of robustness of the modeling and corresponding outputs for goats.

5.4. What hypotheses do the authors have about the disparity between the ivermectin concentrations and the mosquito mortality seen in pigs?

Response: *The variation in 7-day LC50 values across animal species can be attributed to species-specific differences in key factors that influence the pharmacokinetics of ivermectin. These factors include the molecule's volume of distribution, the different body compartments where the molecule is distributed, the*

percentage adipose tissues (since ivermectin is highly hydrophobic), and the metabolism of each species. These parameters quantitatively and qualitatively affect the molecule's distribution across various body compartments, including the blood capillaries in the skin where mosquitoes bite, which in turn affects its bioavailability to mosquitoes and its mosquitocidal efficacy. Due to the species-dependent nature of these parameters, the relationship between ivermectin plasma pharmacokinetics and its actual bioavailability to mosquitoes varies from species to species. Therefore, the effects of ivermectin on mosquito mortality should not be extrapolated or compared across species based solely on plasma concentrations.

In pigs specifically, we observed variability in plasma concentration among animals receiving the same treatment, resulting in differences in efficacy between individual animals. This variability could be attributed to factors such as the animal's weight, physiology (especially adipose percentage), and the injection process itself, which can vary in precision and speed due to the animals' tremors (notably observed in this species), movements, and inconsistencies in human administration.

In line with our reviewer' comment, we thoroughly modified the discussion section, hoping to better address this important question. See ***lines 377-396***

5.5. There is no mention of toxicity in the livestock. Did the authors monitor for toxicity in pigs given three-fold doses?

Response*: All animals from all species were checked daily by cowherds for signs of overdosage and for disease symptoms. No adverse event and no incidence of any disease symptom were noticed during the whole experiment.*

6. **Discussion**

6.1. Please comment in the expected relative densities of each livestock species in the field. Are goats more common than cattle? What order of livestock treatment would you recommend? Cattle > Pigs > Goat > Sheep? Or other?

Response: *Expected relative densities of each of the animal species we considered in our study are now given in the manuscript.*

The treatment order should be based on key parameters specific to the area where the ivermectin-based intervention is planned. These parameters, which must be thoroughly characterized before treatment, include identifying the species present and their blood meal patterns (the realized blood meals), the relative number of alternative hosts compared to humans, and the host species-specific pharmacokinetic/pharmacodynamic (PK/PD) relationship. This also involves determining the duration of significant mortality effects for each mosquito species.

The answer to this remark is given in the new section of the discussion ***lines 451-454 :***

"Therefore, the number of animals to be treated should be determined in consultation with herders, using integrative models to ensure that effectiveness is achieved. Interestingly, not treating entire herds will create refugia for susceptible endo and ectoparasites including Anopheles vectors, providing mitigation strategy against ivermectin resistance [57,58] ».

6.2. There is no mention about the milk or slaughter withdrawal periods and how this may affect deployment of the proposed strategy.

Response: *Indeed, milk or slaughter withdrawal periods could affect deployment, more precisely the treatment coverage, because there will be a significant proportion of the animals left untreated for milk or meat consumption. Benefits for animal health against resources shortage should be evaluated and the number of animals to be treated should be determined in accordance with herders, with the help of integrative models so effectiveness is still reached.*

We took the reviewers comments into account and modified the text accordingly, see **lines 442-451.**

6.3 Please consider mentioning the potential impact of intense treatment schemes or long-lasting formulations on intestinal parasites resistance. What role could refugia play? There is also no mention about the potential long-term theoretical risk of selectin a more anthropophilic mosquito population.

There will necessarily be refugia, as gestating and lactating females, along with animals shortly to be consumed as meat, will not receive the treatment in accordance with Joint FAO/WHO Expert Committee on Food Additives guidelines. Consequently, not treating entire herds will create refuges for susceptible endo and ectoparasites (including Anopheles mosquitoes), which aligns with improved strategies for mitigating antiparasiticide resistance. Similarly, refugia will help reduce selection pressures toward more anthropophilic Anopheles populations. Concerning Anopheles mosquitoes specifically, great refugia are inherently incorporated in the approach since only females will come into contact with ivermectin. Furthermore, the treatment schemes planned to be effective involve transient deployment (before and during the peak transmission season), which may not allow enough time for the selection of resistance traits (physiological or phenotypic) to occur, in endo or ecto-parasites populations.

These notions raised in the 6.2 and 6.3. comments are indeed central to our group's work line toward a sustainable approach and are exposed and discussed in 2 recent articles [55,59]*, the latest being now also cited as a reference in our manuscript. The reviewer's comments are taken into account in our manuscript in a new paragraph* ***lines 447-458:***

"The treatment coverage and overall implementation of the approach will inevitably be constrained by the ivermectin usage guidelines established by the Joint FAO/WHO Expert Committee on Food Additives [56], particularly concerning milk and slaughter withdrawal periods. The benefits to animal health and the long-term wealth of herders must be balanced against potential short-term resource shortages. Therefore, the number of animals to be treated should be determined in consultation with herders, using integrative models to ensure that effectiveness is achieved. Interestingly, not treating entire herds will create refugia for susceptible endo and ectoparasites including *Anopheles* vectors, providing mitigation strategy against ivermectin resistance [57,58]. This same constraint also provides refugia for non-targeted fauna including coprophagic organisms. However, environmental risk assessments should be conducted, and mitigation measures implemented, to ensure the sustainability of this approach and to protect already fragile ecosystems and agro-ecosystems, where manure plays a crucial role in soil fertilization [55,59] ».

Minor

Lines 482. Passed away. Not "are passed away". Also Rest in peace not "rested in peace"

Thank you for the suggestion. The text has been corrected. ***Lines 504-505***
