# Supplementary material for: Impact of blood meals taken on ivermectin-treated livestock on survival and egg production of the malaria vector Anopheles coluzzii under laboratory conditions
Source: PLoS One. 2024 Aug 15;19(8):e0308293. doi: 10.1371/journal.pone.0308293 (PMC11326554; doi:10.1371/journal.pone.0308293)
Supplement: Attachment — Submitted filename: PONE-D-23-39281.docx [file pone.0308293.s007.docx]

**PONE-D-** **23-39281**

Impact of blood meals taken on ivermectin-treated livestock on survival and fecundity of the malaria vector Anopheles coluzzii under laboratory conditions

*This review is by Carlos Chaccour from ISGlobal, Barcelona Institute for Global Health. I have a personal open peer-review policy as the current single-blinded system is riddled with vices.*

This manuscript reports the results of an experiment conducted in Burkina Faso in which pigs, sheep and goats were treated with different doses of ivermectin and then *An. coluzii* mosquitoes were fed on them at different times after treatment. The authors also collected some PK data. The results are discussed on the context of a potential One Health approach to malaria control.

Albeit some minor mistakes, the manuscript is well written. The methods are appropriate for the objectives and the conclusion is supported by the result. I provide here comments for the author`s consideration.

**Introduction**

Consider mentioning early exit as another mosquito behavior contributing to residual transmission. This can be induced by the repellent properties of indoor insecticides or occur even in their absence.

There is no mention of two key concepts: zooprophylaxis vs zoopotentiation.

**Methods**

Please mention the colony`s insecticide resistant status. This is important given the potential cross-metabolic resistance with pyrethroids as they share the same CYP as ivermectin.

Please mention the calculated adipose vs lean weight of the animals.

How was the random allocation of mosquito cups done?

Were cups rotated in the insectary?

Figure 2 is pixelated, making reading it difficult. Additionally, the X axis seems compressed, giving the illusion that the curves are smooth rather than the usual K-M step by step drop. I recommend providing a higher quality image and perhaps even separating it in three different figures to ensure sufficient size. Consider also adding guiding marks to the reader such as the median survival in the control group.

**Results**

The increased gravity found at 28 DAI in goats does not seem to be statistically significant as the CI overlap in figure 3. If that is the case, I recommend, stating it in the text.

The same for the decrease in fecundity reported at day 28 in sheep, or 7 DAI in goats, although in these cases p-values are provided in the text, it is worth it to double check the figure given that CI-overlap.

Table II. The metric should be median and range given the samples come from only two animals.

Can the authors use the PK data to estimate the 7-day LC_50_?

What hypotheses do the authors have about the disparity between the ivermectin concentrations and the mosquito mortality seen in pigs?

There is no mention of toxicity in the livestock. Did the authors monitor for toxicity in pigs given three-fold doses?

**Discussion**

Please comment in the expected relative densities of each livestock species in the field. Are goats more common than cattle? What order of livestock treatment would you recommend? Cattle > Pigs > Goat > Sheep? Or other?

There is no mention about the milk or slaughter withdrawal periods and how this may affect deployment of the proposed strategy.

Please consider mentioning the potential impact of intense treatment schemes or long-lasting formulations on intestinal parasites resistance. What role could refugia play?

There is also no mention about the potential long-term theoretical risk of selectin a more anthropophilic mosquito population.

**Minor**

Lines 482. Passed away. Not “are passed away”. Also Rest in peace not “rested in peace”
